# Lifestyle and perceived health in subjects with chronic bronchitis or emphysema: a cross-sectional study

**DOI:** 10.1186/1471-2458-10-546

**Published:** 2010-09-09

**Authors:** Dejana S Vukovic, Ljudmila M Nagorni-Obradovic, Goran M Vukovic

**Affiliations:** 1 Institute of Social Medicine, School of Medicine Belgrade (Dr Subotica 8), Belgrade (11 000), Serbia; 2 Institute for Lung Disease and Tuberculosis Clinical Centre of Serbia (Pasterova 2) and School of Medicine Belgrade (Dr Subotica 8), Belgrade (11 000), Serbia; 3 Centre for Emergency Surgery Clinical Centre of Serbia (Pasterova 2), Belgrade (11 000), Serbia

## Abstract

**Background:**

The study aim was to compare lifestyle behaviors, body mass index (BMI) and perceived health in subjects with and without chronic bronchitis or emphysema, and to explore if these comparisons differed between demographic subgroups.

**Methods:**

A stratified two-stage sample of the population of Serbia was used; 14.522 adults aged ≥20 years were interviewed.

**Results:**

Compared with controls, respondents with chronic bronchitis or emphysema reported a 23% increased likelihood of eating fresh vegetables every day (CI 1.02-1.48), 58% increased likelihood of currently smoking (CI 1.32-1.88) and more likely to perceive their health as very bad or bad (OR 4.67, CI 3.64-5.98). After stratification for sex, education, and type of settlement, smoking was significantly associated with chronic bronchitis or emphysema in all subgroups except males. The increased likelihood of very bad or bad perceived health in respondents with chronic bronchitis or emphysema was significant in all subgroups, and was highest for respondents ≤65 years of age (adjusted OR 6.51; CI 4.87-8.72) and lowest for respondents >65 years of age (adjusted OR 3.25; CI 2.12-4.97).

**Conclusion:**

Efforts to enhance perceived health and healthy lifestyle behaviors in subjects with chronic bronchitis or emphysema are necessary. Special attention should be paid to smoking cessation in almost all demographic subgroups.

## Background

It is foreseen that respiratory diseases will be growing health problem [1]. Chronic bronchitis and emphysema are significant causes of morbidity in population. Data from the World Health Organization's Large Analysis and Review of European Housing and Health Status Study conducted in random samples from eight European cities showed that in the year preceding (2002-2003) the survey 6.2% of individuals had been diagnosed or treated for chronic bronchitis and emphysema. Furthermore, it was shown that behavioral factors, i.e. physical activities and never smoking were inversely associated with chronic bronchitis and emphysema [2]. Morbidity data for Serbia are incomplete or unreliable. It has been noted that reports contain number of episodes of disease and not actual patients. Data for 2008 show that 2.6% of all deaths in Serbia were caused by chronic obstructive pulmonary disease and moreover, there was an increase of 20.1% in the number of deaths due to obstructive pulmonary diseases between 2002 and 2008 [3].

Chronic bronchitis has been defined in clinical terms as "productive cough that is present for a period of 3 months in each of 2 consecutive years in the absence of another identifiable cause of excessive sputum production" [4]. Emphysema, in contrast, has been defined by its pathological description as "an abnormal enlargement of the air spaces distal to the terminal bronchioles accompanied by destruction of their walls and without obvious fibrosis" [4].

Over the years several different definitions have been offered for COPD. It was described as entity combining chronic bronchitis and pulmonary emphysema, manifested in patients by progressive airflow obstruction with breathing-related symptoms such as chronic cough, exertional dyspnea, expectoration, and wheeze. However, the definition evolved and GOLD in 2001 proposed a definition of COPD that focused on the physiology (progressive nature of airflow limitation), the pathology (airway inflammation), and etiology (noxious particles or gases) [1]. The other important aspect of the new COPD definition is that it was defined spirometrically as airflow limitation that is not fully reversible [4].

It has been demonstrated in various studies that lifestyle behaviors could be associated with progression and outcome of chronic bronchitis and emphysema [2,5-7].

Tobacco smoking has a well-established role in respiratory diseases related morbidity, but less is known about the association between alcohol use and chronic bronchitis and emphysema. Research on the association between alcohol and the morbidity and mortality related to respiratory diseases has produced mixed results. Some studies indicated the positive effects of moderate intake of alcohol on pulmonary function (particularly wine) [8,9], but high consumption (predominantly distilled spirits) has been associated with an independent negative effect on pulmonary function [9].

In recent years, foods rich in antioxidants (fruits, vegetables) have been suggested to protect against obstructive diseases [8,10].

Body mass index (BMI) is closely related to dyspnea score, respiratory-muscle strength, and quality of life in patients with obstructive respiratory diseases [5].

Patients with chronic bronchitis and emphysema are significantly less active than healthy individuals and their activity level is furtherly reduced by acute exacerbations, but regular physical activity is associated with improved pulmonary function in these patients [5,11]. An active lifestyle should be a therapeutic priority in patients with obstructive respiratory diseases because those who carry out some form of regular physical activity have a lower risk of hospital admissions and mortality [5,12].

There is evidence that the risk of developing obstructive respiratory diseases is associated with socio-economic status. This pattern could reflect exposure to indoor and outdoor air pollution, crowding, poor nutrition, occupational exposure or other factors related to low socioeconomic status [6,13-16].

Better understanding of how respondents with chronic bronchitis and emphysema differ from controls without disease, and if these potential differences vary across demographic subgroups, will focus and prioritize future public health interventions for improving the quality-of-life of people with chronic bronchitis and emphysema.

The aim of our study was to compare lifestyle behaviors (smoking, physical activity, fruit and vegetables consumption and alcohol consumption), BMI and perceived health in subjects with and without chronic bronchitis and emphysema in a national, population-based sample, and to explore if these comparisons differ between demographic subgroups (under 65/above 65, urban/rural, elementary, secondary/university education). As making healthy lifestyle choices is important for persons with chronic bronchitis or emphysema, we assumed that those who were aware of their illness would modify their health behaviours accordingly.

## Methods

### Study population

The Institute of Public Health conducted a multipurpose health survey of the population of Serbia (excluding Kosovo) in 2006. A stratified two-stage sample of the population of the Republic of Serbia was used. The sample was selected to provide statistically reliable estimates of health at the national level. Information on the health of the population was obtained from interviews and measurements of anthropometry and blood pressure. Interviews were performed in households of the respondents. Out of 7.673 households selected, 6156 were interviewed. Household response rate was 86.5%. In the households, there were 15.563 adults aged ≥20 years, of which 14.522 were interviewed, yielding a response rate of 93.3%. Overall response rate for adults was 80.5%. All adults aged 20 years and above were included, except those living in institutions.

All respondents were informed about the purpose of the investigation and agreed to participate. The Review Board of the Ministry of Health of Serbia and the Institute of Public Health of Serbia approved the study.

### Measurements

The classification variable was a self-reported history of chronic bronchitis and emphysema as measured by the following question: "Has a doctor ever diagnosed you to have chronic bronchitis or emphysema?" Participants who answered positively were considered to have chronic bronchitis or emphysema. The rest of the sample population served for comparison.

Lifestyle behaviors and perceived health were based on self-reporting and defined as follows. Intake of fruits and vegetables was dichotomized as "eating fresh vegetables (or fresh fruits) every day" or "not eating fresh vegetables (or fresh fruits) every day." (Table 1).

**Table 1:** Questions, answers, and derived variables

Question	Answers	Derived variables
Has a doctor ever diagnosed you to have chronic bronchitis or emphysema	Yes	Having chronic bronchitis/emphysema
	No	Not having chronic bronchitis/emphysema
		
How often, during the past week, have you eaten:	Not once	Less than every day
Fruits	1-2 times	
	3-5 times	
	6-7 times	Every day
		
How often, during the past week, have you eaten: Vegetables	Not once	Less than every day
	1-2 times	
	3-5 times	
	6-7 times	Every day
		
Have you ever smoked in your life?	Yes	Those who never smoked- **non smokers**
	No	
Have you smoked at least 100 cigarettes in your life?	Yes	
	No	Those who used to smoke but do not smoke now - **ex smokers**
Do you smoke now?	Yes, every day	Those who smoke occasionally or every day - **smokers**
	Yes, from time to time	
	No	
		
Which of the following statements refer to you?	Never consumed before.	Not heavy (not at risk)
	Tried drinking once or twice	
	Used to but not any more	
	I drink occasionally.	
	I drink every day	Heavy (at risk)
		
How often does it happen that you drink 6 or more alcoholic drinks on a single occasion?	Never	Not at risk
	Less than once a month	
	Once a month	At risk
	Once a week	
	Once a day or almost every day	
		
If we look back on the past year, what would you say best describes your spare time activities?	Hard training and competitive sports	Active
	Recreational sports or heavy gardening	
	Walking, bicycling or other light activities at least 4 hours a week	
	Reading, watching TV or other sedentary activity	Not active
		
How would you rate your health in general?	Very bad	Very bad/bad
	Bad	
	Fair	Fair
	Good	Very good/Good
	Very good	

Smoking was dichotomized as "current smoker" versus "nonsmoker". Respondents who reported smoking every day or some days, and consuming ≥100 cigarettes in their lifetime were classified as "current smokers". Nonsmokers included former smokers (i.e., smoked ≥100 cigarettes in their lifetime, but did not currently smoke) and "never smokers".

At risk for binge alcohol use was defined as having ≥6 drinks on one occasion at least once a month. Respondents at risk for heavy consumption (yes/no) were defined as having any type of alcohol every day (Table 1).

Questions about physical activities in leisure time (yes/no) were asked to assess sedentary behavior. Respondents who mainly read, watched television or had similar activities in their leisure time were classified as "physically not active". Those who walked, cycled, ran, swam or did gardening for at least 4 hours per week were classified as "physically active".

Weight and height were measured by trained health workers following a defined protocol. BMI was calculated based on these values. Respondents were categorized as normal (BMI <25), overweight (25 to 30) or obese (> 30) (Table 1).

Perceived health was assessed by the question "How would you rate your health in general?" A five-point scale was used, and answers were categorized into very bad/bad; fair; or good/very good (Table 1).

Potential confounding demographic factors (age, sex, education, type of settlement, wealth index) were also considered.

### Statistical analyses

Weighted prevalence estimates of lifestyle behaviors, BMI, perceived health and demographic factors for respondents with and without chronic bronchitis and emphysema were calculated with differences examined by chi-square test for categorical variables and t-test for continuous variables.

The relationship between the history of chronic bronchitis and emphysema diagnosis and lifestyle behaviors, BMI, and perceived health were examined with unadjusted and adjusted prevalence odds ratios (OR) and 95% confidence interval (CI) calculated using multivariable logistic regression. Adjustment was made for sex, age in years (continuous), education (elementary, secondary, university) and type of settlement (urban/rural). Associations between chronic bronchitis and emphysema diagnosis and lifestyle behaviors, BMI, and perceived health were assessed with multivariable logistic regression calculation of adjusted ORs for demographic subgroups. Each demographic characteristic (age, sex, education, type of settlement) was dichotomized (age: ≤65/> 65; sex: male/female; education: elementary/secondary, university; settlement: urban/rural). Only two subgroups were considered for each characteristic to facilitate adequate stratum-specific sample sizes. For the subgroup analysis, ORs were adjusted for the other three demographic characteristics. Specifically, ORs for age subgroups (i.e., ≤65 and >65 years of age) were adjusted for sex, education, and type of settlement. ORs for sex subgroups were adjusted for age, education, and type of settlement; ORs for education subgroups were adjusted for age, sex, and type of settlement; and ORs for type of settlement subgroups were adjusted for sex, age and education.

The health survey of the population of Serbia was supported and financed by the Ministry of Health of the Republic of Serbia.

## Results

Weighted prevalence for demographic factors is included in Table 2. Weighted prevalence for lifestyle behaviors, BMI, perceived health are presented in Table 3. Respondents with chronic bronchitis or emphysema were significantly older (mean age, 57.98 ± 17.65 years) compared with controls (50.72 ± 17.95 years). Significant differences between respondents with chronic bronchitis or emphysema and controls were found also for sex (37.7% males compared with 44.4% males among controls), education (51.3% of those with elementary education among persons with chronic bronchitis or emphysema compared with 40.6% among controls) and wealth index (among persons with chronic bronchitis or emphysema there were 26.5% pertaining to the poorest quintile compared with 22.2% among controls). No significant difference was found by settlement type.

**Table 2:** Weighted prevalence estimates of demographic factors among respondents with chronic bronchitis or emphysema and controls

	Respondents with bronchitis or emphysema	Controls	P
Age (%)			
20-29 years	11.0	15.6	0.000
30-39 years	6.1	15.7	
40-49 years	12.4	16.1	
50-59 years	18.4	18.3	
60-69 years	17.4	13.9	
70+ years	34.7	20.1	
Mean ± SD (years)	57.98 ± 17.65	50.72 ± 17.95	0.000
			
Sex (%)			
Female	62.3	55.6	0.000
Male	37.7	44.4	
			
Education (%)			
Elementary	51.3	40.6	0.000
Secondary	39.4	47.2	
University	9.4	12.2	
			
Type of settlement (%)			
Urban	48.0	48.2	0.904
Other	52.0	51.8	
			
Wealth index (%)			
Poorest	26.5	22.2	0.022
Second quintile	23.1	21.8	
Third quintile	19.0	21.7	
Fourth quintile	17.3	18.3	
Richest	14.1	15.9	

**Table 3:** Weighted prevalence estimates of lifestyle behaviors, BMI and perceived health among respondents with chronic bronchitis or emphysema and controls and Mean ± SD of BMI

	Respondents with bronchitis or emphysema N (%)	Controls N (%)	P
Consumption of fruits			
Every day	313 (39.6)	6019 (44.3)	0.008
Less than every day	474 (60.4)	7619 (55.7)	
			
Consumption of vegetables			
Every day	433 (56.5)	7385 (55.4)	0.521
Less than every day	333 (43.5)	5946 (44.6)	
			
Smoking			
Current smokers	301 (38.8)	4732 (35.4)	0.042
Ex-smokers	148 (19.1)	1905 (14.2)	
Nonsmokers	327 (42.1)	6730 (50.4)	
			
Binge alcohol use			
At risk	29 (3.9)	580 (4.4)	0.431
Not at risk	721 (96.1)	12591 (95.6)	
			
Heavy consumption of alcohol			
At risk	32 (4.2)	606 (4.6)	0.566
Not at risk	718 (95.8)	12565 (95.4)	
			
Physical activity			
Active	190 (25.2)	3370 (29.9)	0.004
Not active	563 (74.8)	7901 (70.1)	
			
Body mass index			
< 25	316 (42.0)	5670 (45.1	0.000
25-30	258 (34.3)	4513 (35.9	
> 30	178 (23.7)	19.0 (2389)	
Mean ± SD	27.08 ± 9.85	26.64 ± 11.66	-2.671, 0.008
			
Perceived health			
Very bad/bad	290 (38.4)	2123 (16.6)	0.000
Fair	316 (41.9)	4859 (38.0)	
Good/very good	147 (19.6)	5806 (45.4)	

Fruit consumption, smoking and physical activity demonstrated statistically significant differences for respondents with chronic bronchitis or emphysema versus controls. More than a half of respondents ate vegetables every day, and there was no significant difference between respondent with chronic bronchitis or emphysema and those without. Among respondents with chronic bronchitis or emphysema, 39.6% ate fresh fruits daily compared with 44.3% among controls. Respondents with chronic bronchitis or emphysema were significantly more likely to be current smokers than controls (38.8% vs. 35.4%) and less likely to be physically active than controls (25.2% vs. 29.9%). Regarding alcohol consumption there was no significant difference in binge drinking or heavy alcohol consumption between respondents with disease and controls. Respondents with chronic bronchitis or emphysema were less likely to perceive their health as very good/good (19.6% vs. 45.4%) and more likely to be obese (23.7% vs. 19.0%) [3].

Adjusted and unadjusted ORs for associations between history of chronic bronchitis or emphysema diagnosis and lifestyle behaviors, BMI, and perceived health are presented in Table 4. In the logistic regression model prevalence of a regular fruit consumption, heavy or binge alcohol use, physical activity, and BMI was not significantly associated with chronic bronchitis or emphysema; but significant association was found with eating daily fresh vegetables (OR 1,22, CI 1.02-1.48), being current smoker (OR 1.58, CI 1.32-1.88, with reporting very bad or bad health (OR 4.67; CI 3.64-5.98), and fair health (OR 2.17, CI 1.75-2.68).

**Table 4:** Unadjusted and adjusted prevalence odds ratio (OR) and 95% confidence interval (CI) for the associations between chronic bronchitis/emphysema diagnosis and lifestyle behaviors, body mass index, and perceived health (N = 790)

Lifestyle/health behavior	Unadjusted OR (95% CI)	Adjusted OR (95%CI)*
Consumption of fruits		
Every day	0.955 (0.794-1.149)	0.941 (0.780-1.135)
Less than every day	1.00	1.00
		
Consumption of vegetables		
Every day	1.221 (1,013-1.472)	1.229 ((1.018-1.482)
Less than every day	1.00	1.00
		
Smoking		
Current smokers	1.341 (1.134-1.585)	1.579 (1.321-1.886)
Nonsmokers	1.00	1.00
		
Binge alcohol use		
At risk	0.958 (0.644-1.426)	1.139 (0.759-1.707)
Not at risk	1.00	1.00
		
Heavy consumption of alcohol		
At risk	1.064 (0.736-1.540)	1.035 (0.706-1.516)
Not at risk	1.00	1.00
		
Physical activity		
Active	0.990 (0.826-1.187)	1.067 (0.88-1.282)
Not active	1.00	1.00
		
Body mass index		
< 25	1.00	1.00
25-30	0.940 (0.781-1.132)	0.907 (0.752-1.094)
> 30	1.073 (0.869-1.325)	1.006 (0.814-1.244)
		
Perceived health		
Very bad/bad	6.171 (4.921-7.739)	4.667 (3.643-5.977)
Fair	2.577 (2.099-3.163)	2.168 (1.753-2.682)
Good/very good	1.00	1.00

• Adjusted for sex, age in years, education, and urban living environment. 1.00 reference category

Adjusted ORs stratified by age, sex, education, and type of settlement are presented in Table 5 to explore potential differences in the associations across demographic subgroups between lifestyle behaviors, BMI, and perceived health in respondents with chronic bronchitis or emphysema and controls.

**Table 5:** Adjusted prevalence odds ratio (OR) and 95% confidence interval (CI) for the association between diagnosis of chronic bronchitis/emphysema and lifestyle behaviors, body mass index, and perceived health stratified by age, sex, education, and urban living environment

		Age*		Sex#		Education¶		Settlement†	
	
		≤65	> 65	male	female	elementary	Secondary/university	urban	rural
Consumption of fruits	Every day	1.031 (0.816-1.304)	0.781 (0.572-1.067)	0.757 (0.559-1.027)	1.071 (0.842-1.363)	0.937 (0.706-1.244)	0.941 (0.733-1.207)	0.916 (0.699-1.200)	0.950 (0.733-1.233)
	Less than every day	1.00	1.00	1.00	1.00	1.00	1.00	1.00	1.00
Consumption of vegetables	Every day	1.092 (0.862-1.384)	1.548 (1.139-2.104)	1.257 (0.938-1.686)	1.214 (0.950-1.551)	1.294 (0.983-1.704)	1.170 (0.905-1.512)	1.188 (0.907-1.556)	1.278 (0.984-1.661)
	Less than every day	1.00	1.00	1.00	1.00	1.00	1.00	1.00	1.00
Smoking	Current smokers	1.548 (1.259-1.904)	1.692 (1.191-2.403)	1.270 (0.960-1.680)	1.801 (1.429-2.269)	1.563 (1.156-2.112)	1.579 (1.263-1.974)	1.658 (1.297-2.120)	1.491 (1.149-1.935)
	Nonsmokers	1.00	1.00	1.00	1.00	1.00	1.00	1.00	1.00
Binge alcohol use	At risk	1.244 (0.788-1.964)	0.896 0.368-2.186)	0.949 (0.583-1.545)	2.294 (1.106-4.757)	1.387 (0.734-2.619)	1.014 (0.597-1.722)	1.010 (0.558-1.830)	1.279 (0.733-2.231)
	Not at risk	1.00	1.00	1.00	1.00	1.00	1.00	1.00	1.00
Heavy consumption of alcohol	At risk	0.863 (0.482-1.545)	1.183 (0.705-1.983)	1.068 (0.710-1.606)	0.748 (0.221-2.530)	0.959 (0.558-1.649)	1.110 (0.643-1.915)	1.059 (0.566-1.981)	1.009 (0.620-1.644)
	Not at risk	1.00	1.00	1.00	1.00	1.00	1.00	1.00	1.00
Physical activity	Active	1.051 (0.846-1.307)	1.071 (0.760-1.511)	1.217 (0.920-1.610)	0.951 (0.743-1.217)	1.088 (0.809-1.465)	1.048 (0.829-1.324)	1.207 (0.933-1.561)	0.947 (0.726-1.235)
	Not active	1.00	1.00	1.00	1.00	1.00	1.00	1.00	1.00
Body mass index	< 25	1.00	1.00	1.00	1.00	1.00	1.00	1.00	1.00
	25-30	0.942 (0.742-1.194)	0.969 (0.713-1.318)	0.783 (0.585-1.049)	1.006 (0.785-1.289)	0.945 (0.705-1.267)	0.867 (0.677-1.110)	0.810 (0.616-1.064)	1.022 (0.788-1.327)
	> 30	1.128 (0.862-1.477)	0.967 (0.679-1.377)	1.073 (0.757-1.520)	0.993 (0.755-1.306)	1.196 (0.880-1.627)	0.820 (0.602-1.116)	0.926 (0.678-1.264)	1.115 (0.831-1.495)
Perceived health	Very bad/bad	6.514 (4.868-8.717)	3.252 (2.126-4.973)	4.045 (2.790-5.863)	5.434 (3.888-7.594)	4.359 (2.959-6.421)	4.833 (3.455-6.762)	4.590 (3.210-6.562)	4.811 (3.398-6.810)
	Fair	2.459 (1.929-3.136)	1.839 (1.224-2.765)	1.752 (1.269-2.419)	2.520 (1.892-3.356)	2.019 (1.385-2.944)	2.214 (1.710-2.866)	2.144 (1.596-2.879)	2.240 (1.645-3.050)
	Good/very good	1.00	1.00	1.00	1.00	1.00	1.00	1.00	1.00

*adjusted for sex, education and settlement; # adjusted for age, education and settlement; ¶ adjusted for age, sex and settlement; †adjusted for age, sex and education; 1.00 reference category

Variables with non-significant ORs in table 4 before stratification (regular fruit consumption, binge and heavy alcohol use, physical activity, BMI) did not demonstrate significant association in certain subgroups after stratification, except for binge alcohol use in females. The binge alcohol use was significantly associated in females with bronchitis or emphysema (adjusted OR 2.29; CI 1.11-4.76).

Vegetable consumption adjusted were significantly associated with chronic bronchitis or emphysema but, after stratification, only those aged >65 years demonstrated a significantly increased likelihood of consuming fresh vegetables daily (adjusted OR 1.55; CI 1.14-2.10). After stratification, smoking was significantly associated with chronic bronchitis or emphysema in all subgroups except in males. The increased likelihood of very bad or bad perceived health in respondents with chronic bronchitis or emphysema was significant in all subgroups, and was highest for respondents aged ≤65 years (adjusted OR 6.51; CI 4.87-8.72) and lowest for respondents aged >65 years (adjusted OR 3.25; CI 2.12-4.97).

## Discussion

Various studies evaluated individual aspects of lifestyle in patients with chronic bronchitis or emphysema, but we analyzed multiple lifestyle behaviors. Our study was based on a national sample and compared people with and without chronic bronchitis or emphysema.

Based on a national health survey, subjects with chronic bronchitis or emphysema differ from those without these diseases with regard to fruit consumption, smoking, physical activity, BMI and perceived health. When adjusted for sex, age, education, and type of settlement, ORs were significant only for consumption of vegetables, smoking and perceived health. Demographic characteristics may influence the prevalence of lifestyle behaviors among subjects with chronic bronchitis or emphysema.

With regard to lifestyle behaviours, regular consumption of vegetables was significantly associated with chronic bronchitis or emphysema. This is an important finding because some studies have demonstrated beneficial effects of fruits and vegetables on respiratory function [10]. It may be partly explained by the fact that diagnosis of a serious chronic disease may cause an individual to adopt healthier behaviors. Our study was cross-sectional and we could not establish time since diagnosis, so we cannot postulate the cause-effect relationship. After data were disaggregated by demographic variables, only in group of respondents older than 65 the vegetable consumption was significantly associated with chronic bronchitis or emphysema. We expected a lower prevalence of current smokers among respondents with the disease because smoking cessation is a highly recommended measure for treatment of patients with obstructive respiratory diseases [1,17]. Our results showed a significantly higher prevalence and a significant association of smoking in respondents with the disease in total sample and in all demographic subgroups except males. Such results could indicate that insufficient attention was paid to smoking cessation programs for patients with chronic bronchitis or emphysema and it suggests the importance of smoking as a risk factor for obstructive respiratory diseases. It has been noticed that studies concerning women's health problems in this field and epidemiological studies of lung function impairment in women and risk factors in a long-term perspective are rare. However, some authors suggest that life-style factors such smoking among women, are related to airway symptoms and also quality of life even many years later [16].

In females, the likelihood of binge alcohol use was significantly associated with the presence of the disease. Controlling alcohol consumption could be important for patients with chronic bronchitis or emphysema (particularly women) because excessive consumption of alcohol is associated with negative effects on pulmonary function [7,9].

Our finding did not show significant association of physical activity, regular fruit consumption and BMI and presence of chronic bronchitis or emphysema in none of analysed population subgroups. However, it has been demonstrated that increased intake of food rich in antioxidants, like fruits could improve prognosis of persons with obstructive respiratory diseases [8,10,18].

After adjustment for sex and age, no significant association of regular physical activity with the presence of chronic bronchitis or emphysema was found, which is different from other studies that showed significantly lower physical activity among persons with these diseases [11]. In our study great majority of those with and without the disease were physically inactive. Therefore, actions to promote active lifestyle among patients with chronic bronchitis or emphysema should be recommended [12].

Our findings suggest a strong association of bad or very bad perceived health among respondents with chronic bronchitis or emphysema compared with respondents without the disease: this is consistent with other studies [19,20]. Studies found that poor health self-perception was associated with the female sex [21,22]. Our analyses showed that poor self-rated health was expressed more in women with chronic bronchitis or emphysema than in men.

Limitations of our study are related to measurement bias because diagnosis of chronic bronchitis or emphysema and lifestyle behaviors was reported by the respondent. Self-reported diagnosis of chronic bronchitis or emphysema could underestimate prevalence of the disease because mild and moderate stages could be undiagnosed, or subjects could not have consciousness of their disease above all in case they are smokers; in fact smokers tend to attribute obstructive respiratory symptoms and, exacerbations to smoking effects. However, questionnaires were found to be valid instrument for population research for respiratory diseases [23]. Recently published paper emphasized the importance of population studies based on questionnaires and their significance for global respiratory illness surveillance for prevention, health policy and management [24]. Prospective cohort studies are needed to assess temporal changes in lifestyle behaviors, BMI, and perceived health that occur after the diagnosis of chronic bronchitis or emphysema.

## Conclusions

Our study provided results based on a large population sample and revealed health related behaviours among persons with chronic bronchitis or emphysema. Results showed that even when being aware of having a chronic respiratory disease and perceiving their health worse than the rest of the population, persons with chronic bronchitis or emphysema still practice certain unfavorable behaviours. Although making healthy lifestyle choices is important for persons with chronic bronchitis or emphysema, the prevalence of healthy behaviours is less than optimal regarding smoking, diet and physical activity. Efforts to enhance perceived health and healthy lifestyle behaviors among subjects with chronic bronchitis or emphysema are necessary. Special attention should be paid to smoking cessation in almost all demographic subgroups. Certain demographic subgroups need more

Focused intervention, such as addressing binge drinking among women with chronic Bronchitis or emphysema, as well as addressing poorer perceived health in subgroups aged <65 years.

Prospective cohort studies are needed to assess changes in lifestyle behaviors, BMI, and Perceived health that occurs after the diagnosis of chronic bronchitis or emphysema.

## Competing interests

The authors declare that they have no competing interests.

## Authors' contributions

DV participated in planing and designing of the study, data acquisition, analysis of the data and drafted the article. LNO participated in planing and designing of the study, analysing and interpreting the results and drafting the article. GV participated in interpreting and presenting the results and drafting the article. All authors read and approved the final manuscript.

## Pre-publication history

The pre-publication history for this paper can be accessed here:

http://www.biomedcentral.com/1471-2458/10/546/prepub
